# Bioinformatic Identification of miR-622 Key Target Genes and Experimental Validation of the miR-622-RNF8 Axis in Breast Cancer

**DOI:** 10.3389/fonc.2019.01114

**Published:** 2019-10-23

**Authors:** Chuanyang Liu, Lu Min, Jingyu Kuang, Chushu Zhu, Xin-Yuan Qiu, Lingyun Zhu

**Affiliations:** Department of Biology and Chemistry, College of Liberal Arts and Sciences, National University of Defense Technology, Changsha, China

**Keywords:** breast cancer, bioinformatics, miRNA targets, miR-622-RNF8 axis, EMT

## Abstract

Breast cancer is the leading cause of cancer-associated deaths among females. In recent decades, microRNAs (miRNAs), a type of short non-coding RNA that regulates gene expression at the post-transcription level, have been reported to participate in the regulation of many hub genes associated with tumorigenesis, tumor progression, and metastasis. However, the precise mechanism by which miRNAs regulate breast cancer metastasis remains poorly discussed, which limits the opportunity for the development of novel, effective therapeutic targets. Here, we aimed to determine the miR-622-related principal regulatory mechanism in cancer. First, we found that miR-622 was significantly related to a poor prognosis in various cancers. By utilizing an integrated miRNA prediction process, we identified 77 promising targets and constructed a protein-protein interaction network. Furthermore, enrichment analyses, including GO and KEGG pathway analyses, were performed to determine the potential function of miR-622, which revealed regulation networks and potential functions of miR-622. Then, we identified a key cluster comprised of six hub genes in the protein-protein interaction network. These genes were further chosen for pan-cancer expression, prognostic and predictive marker analyses based on the TCGA and GEO datasets to mine the potential clinical values of these hub genes. To further validate our bioinformatic results, the regulatory axis of miR-622 and RNF8, one of the hub genes recently reported to promote breast cancer cell EMT process and breast cancer metastasis, was selected as *in vitro* proof of concept. *In vitro*, we demonstrated the direct regulation of RNF8 by miR-622 and found that the predicted miR-622-RNF8 axis could regulate RNF8-induced epithelial-mesenchymal transition, cell migration, and cell viability. These results were further demonstrated with rescue experiments. We established a closed-loop miRNA-target-phenotype research model that integrated the bioinformatic analysis of the miRNA target genes and experimental validation of the identified key miRNA-target-phenotype axis. We not only identified the hub target genes of miR-622 *in silico* but also revealed the regulatory mechanism of miR-622 in breast cancer cell EMT process, viability, and migration *in vitro* for the first time.

## Introduction

Breast cancer (BC) is the common form of malignant cancer and the primary cause of cancer-associated deaths among females (1–3). In 2018, it was estimated that ~40 thousand people died of breast cancer, accounting for 14% of cancer-associated deaths in America (3). Although great progress has been made in diagnosis and therapeutic methods, the high incidence and mortality rate of breast cancer indicate the necessity for developing novel therapeutic strategies (4). To address this issue, it is essential to elucidate the molecular mechanisms underlying the pivotal processes of breast cancer, such as tumorigenesis and metastasis (5, 6). Epithelial-mesenchymal transition (EMT) have been shown to play a role in these tumorigenic processes (7). During the EMT process, epithelial cells lose their cell-to-cell junctions and are converted into non-polarized and motile mesenchymal cells, acquiring the abilities to resist apoptosis, disseminate and invade into the blood and lymphatic vessels, which ultimately form small metastases and lead patients to multiple organ failure and death (8–11). Some molecular signatures with significantly altered expression in the EMT process are regarded as hallmarks of EMT, including the downregulation of epithelial markers, including E-cadherin and ZO-1, and the upregulation of mesenchymal markers, including Snail, N-cadherin, and fibronectin (12–15). In light of the importance of EMT in tumorigenesis and metastasis, there is a clear need to improve our understanding of the molecular mechanism underlying this essential biological process (15, 16).

Mi(cro)RNAs are a class of small RNAs originally transcribed from non-coding regions, ~19 to 24 nucleotides in length (16–19). Since their discovery in 1993, miRNAs have been demonstrated to play critical roles in the posttranscriptional regulation of gene expression (17, 18). By base-pairing to miRNA recognition elements (MERs) located in the 3'-untranslated regions (3' UTRs) of target mRNAs, mature miRNAs induce posttranslational repression or mRNA degradation of the target gene (20). It has been estimated that over 30% of all protein-coding genes in humans are regulated by miRNAs, indicating that miRNA regulation might be the most abundant mode of posttranscriptional regulation (21–24). Recently, a large body of work has demonstrated the important roles of miR-622 in regulating cancer pathogenesis. Studies have demonstrated that the expression of miR-622 is downregulated in gastric cancer, and the overexpression of miR-622 can inhibit cell invasion and tumor metastasis by targeting ING1 (inhibitor of growth family, member 1) (25). Subsequently, the downregulation of miR-622, which regulates cancer progression by targeting corresponding target genes, was validated in a series of cancers, such as bronchogenic carcinoma (26), lung cancer (27), and liver cancer (26, 28). Interestingly, it has been found that the elevated expression of miR-622 is positively correlated with a poor prognosis in cancer patients during anticancer treatment. In ovarian cancer, it has also been reported that the overexpression of miR-622 leads to platinum chemotherapy resistance (29). miR-622 can also directly target transcription factor 2 (ATF2), which acts as both an oncogene and a tumor suppressor in tumorigenesis. These studies provide a specific explanation by which miR-622 regulates a specific phenotype via a single target. However, in a larger landscape, how miR-622 regulates interaction networks in cells and how miR-622 target axes regulate phenotype are poorly discussed. The identification of miR-622-targeting hub genes would provide deeper insight into the mechanism underlying miR-622-induced downstream phenotype alterations.

**Figure 1:**
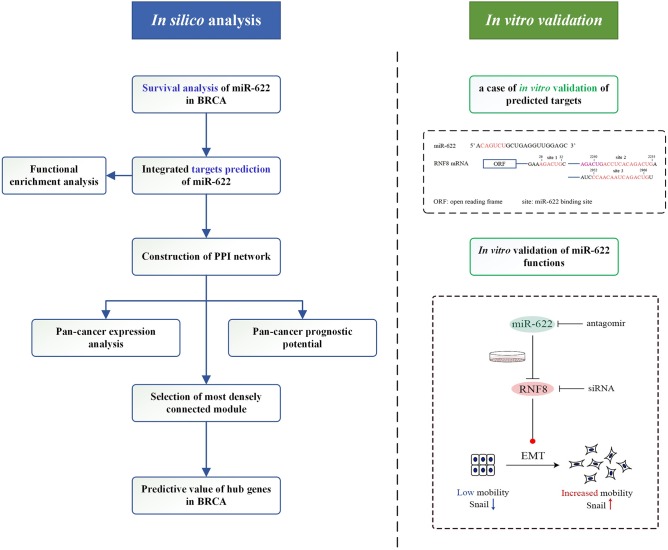
Graphical representation of the workflow in the present study.

In this study, we aimed to determine the main molecular mechanism and functions of miR-622. We designed a workflow to explore the function of miR-622-target axes by integrating *in silico* analyses and *in vitro* validation (Figure 1). We first explored the potential prognostic value of miR-622 in various cancers by Kaplan-Meier survival analysis in different databases, including the GEO and the TCGA. To understand the principal regulatory pattern of miR-622, we predicted the targets of miR-622 by an integrated targeting prediction. In total, 77 overlapping genes were chosen, and Gene Ontology (GO) and pathway enrichment analyses were further performed with these overlapping targets. Using the STRING database, we constructed a protein-protein interaction network with 25 nodes to reveal a potential regulatory model among these overlapping genes. Pan-cancer expression and survival analyses were performed on these 25 genes, and we found that 17 genes could be used as potential prognostic markers of breast cancer. With the MCODE module in Cytoscape software, a key cluster with six genes was further chosen as hub genes. The predictive values of the hub genes were further explored to evaluate their prognostic and predictive potential. Our results showed that HIST2H2BE, DYRK2, MBD2, and RB1 could be used as predictive markers for breast cancer.

Through a review of miR-622-related reports, we found that some of our predicted hub target genes were already experimentally validated as targets of miR-622, indicating the value and correctness of our work. To further support our hypotheses *in vitro*, we chose breast cancer, in which the role of miR-622 has never been studied previously, and RNF8, one of six hub genes identified previously, as proof of the concept of our bioinformatic analysis. We designed complete *in vitro* experiments and validated the regulatory pattern of miR-622-RNF8 in breast cancer EMT process because the relationship between RNF8 and breast cancer metastasis (including EMT) has been demonstrated recently (12). We first verified the direct binding and regulation of RNF8 by miR-622. Subsequently, we demonstrated that miR-622 can regulate the EMT process, cell migration, and cell viability in breast cancer by directly regulating RNF8 expression. Through *in vitro* experiments, we found that the downregulation of miR-622 by an antagomir promoted breast cancer cell migration, while the upregulation of miR-622 inhibited cell migration. Rescue experiments further demonstrated that miR-622-induced phenotypes, such as *in vitro* migration and EMT marker changes, were reversed by the overexpression of RNF8 in the same tumor cells, highlighting the regulatory function of the miR-622-RNF8 axis on breast cancer cells and further proving the identified hub targets of miR-622.

## Methods

### Evaluation of the Prognostic Value of miR-622

#### Pan-Cancer Survival Analysis of miR-622 in GEO and TCGA Dataset

Pan-cancer overall survival analysis based on pan-cancer TCGA miRNA database were analyzed by Kaplan-Meier Plotter (http://kmplot.com/analysis/) with 21 kinds of cancers. In breast cancer, especially, we downloaded the survival data of breast cancer tissue METABRIC (*n* = 1262), TCGA (*n* = 1077, BRCA). The R package “survival” and “survminer” was used for survival analysis and visualization.

### Prediction of miR-622 Target Genes

#### Integrated Target Prediction of miR-622

MiRWalk 3.0 (http:// http://mirwalk.umm.uni-heidelberg.de/), an easily accessible database including predicted data obtained with a machine learning algorithm, its focus lies on accuracy, simplicity, user-friendly design, and mostly up to date information (30). We analyzed the potential miR-622 target genes with this database and four other highly recognizable and promising miRNA-target prediction tools (miRanda: http://www.microrna.org/microrna/getMirnaForm.do, miRDB: http://mirdb.org/, RNA22: https://cm.jefferson.edu/rna22/ and Targetscan7.2: http://www.targetscan.org/vert_72/). The miR-622 target genes predicted by both five prediction programs were identified for further analysis. The relationships between these targets' sets were analyzed and visualized by bioinformatic software TBtools and R package “UpSetR” (31, 32).

### Prediction of miR-622 Target Genes

#### Functional Enrichment Analysis

The selected 77 overlapping genes were then deposited to the Metascape (metascape.org/gp/index.html) for further GO annotation and KEGG pathway enrichment analysis. Metascape database offers an online tool for gene annotation and analysis resource that assists biologists to make sense of one or multiple gene lists (33). In the present study, the Metascape database was applied to investigate GO annotation and KEGG pathways of overlapping genes. *P* < 0.05 was considered as significant. Furthermore, Reactome (*P* < 0.01), KEGG disease (*P* < 0.05), NGHRI_GWAS_Catalog (*P* < 0.05), and PATHER pathway (*P* < 0.01) enrichment analysis were also performed by KOBAS database. R language were used to visualized the results of GO annotation and KEGG pathway enrichment analysis. The predicted targets of miRWalks 3.0 (with score>0.95 and binding sites 3'UTR) were also submit to its own GO annotation and KEGG pathway enrichment analysis to avoid the key information missing after only considering the intersection of predicted targets.

#### Protein-Protein Interaction Analysis

Protein-protein interaction (PPI) network of overlapping genes was construct by the Search Tool for the Retrieval of Interacting Genes (STRING, https://string-db.org/). STRING database is an online and user-friendly database resource with integrated information of interactions of proteins from prediction or experiments. In present study, medium confidence (minimum required interaction score>0.400) was the selection criterion to construct the PPI network, disconnected nodes were excluded from the network. The list of PPI pairs was downloaded for further analysis and visualized by Cytoscape software (version 3.7.1). Molecular Complex Detection (MCODE) plugin in Cytoscape was utilized to find potential cluster in the PPI network based on topology, which may help identify the most likely key target genes for miR-622. The degree cut-off value to 2 and the node score cut-off to 0.2 were set in the MCODE process.

### Prediction of miR-622 Target Genes

#### Pan-Cancer Expression of Overlapping Genes

In order to explore the expression pattern of overlapping genes in cancer, we utilized the Gene Expression Profiling Interactive Analysis (GEPIA) tool (http://gepia.cancer-pku.cn/) to compare the expression of overlapping genes in cancers and their corresponding normal tissue. GEPIA integrated mRNA sequencing data from TCGA and the Genotype-Tissue Expression (GTEx) project, providing customizable functionalities for differential expression analysis, profiling plotting, patient survival analysis, and so on (34, 35). The pan-cancer expression of overlapping genes was shown as heatmap using R package “pheatmap.”

#### Pan-Cancer Prognostic Value of Overlapping Genes

In order to explore the prognostic of overlapping genes in breast cancer, we firstly analyzed the overall survival rate of these genes in breast cancer using Kaplan-Meier Plotter (http://kmplot.com/analysis/) microarray dataset (1,402 samples), JetSet best probe set was used to represent each gene with gene expression auto best cut-off (36, 37). To further assess the prognostic value of these genes, GEPIA tool (http://gepia.cancer-pku.cn/), including integrated TCGA mRNA sequencing data and the GTEx, were also used (with FDR P-value adjustment, 0.05 significance level and Median group cut-off) to calculate patient OS (34, 35). The results were shown in form of heatmap with colors of cells showing log_10_(HR) and the frame meaning significance.

#### Predictive Value of Hub Genes

Six genes, clustered by MCODE, were selected as hub target genes of miR-622. The ROC plotter, the first online transcriptome-level validation tool for predictive biomarkers, was utilized to assess the predictive potential of these six hub genes (38). JetSet best probe set was used to represent each gene with gene expression auto best cut-off (37). Data were downloaded from ROC plotter and analyzed by Graphpad Prism 8.0. each gene was compared between non-responding or responding group to any kind of chemotherapy including Taxane, Anthracycline, lxabepilone, CMF, FAC, and FEC *p* < 0.05 was considered as significance. Receiver operator characteristic curve (ROC) were used to assess the predictive roles of hub genes.

### Experimental Validation of miR-622/RNF8 Regulation Axis

#### Cell Culture

Breast cancer cell lines MDA-MB-231, MCF7, and human embryonic kidney cell line 293T were purchased from American Type Culture Collection (ATCC, Manassas, VA), and cultured in RPMI 1640 Medium (Hyclone) supplemented with 10% fetal bovine serum (FBS; GIBCO, Gaithersburg, MD, USA) and 100 U/ml penicillin and streptomycin (P/S; Hyclone). Cells were contained in a 5% CO_2_ incubator at 37°C.

#### Overexpression or Knockdown of miRNA and RNF8

MiR-622 overexpression and knockdown were performed by liposome-mediated miRNA Agomir and Antagomir transfection. Chemically modified Hsa-miR-622 mimics (miR-622 Agomir, Ago, 5'-ACAGCAGGCACAGACAGGCAGU-3') and hsa-miR-622 inhibitor (miR-622 Antagomir, Anti, 5'-ACUGCCUGUCUGUGCCUGCUGU-3'), with their negative control Stable N.C (Sta.N.C, 5'-UUCUCCGAACGUGUCACGUTT-3'), and inhibitor N.C (Inh.N.C, 5'-CAGUACUUUUGUGUAGUACAA-3'), respectively, were purchased from GenePharma. RNF8 siRNA (si-RNF8-1, 5'-GGACAAUUAUGGACAACAA-3') was purchased from GenePharma. MDA-MB-231, MCF7, and HEK293T cells were transfected using Lipofectamine 2000 (Invitrogen) according to the manufacturer's directions with the working concentration of Antagomir/Agomir 20 nM, siRNA 30 nM.

#### RNA Extraction and Quantitative Real-Time PCR

Total RNAs were extracted using the TRIzol agent (Ambion) according to the instruction of the manufacturer. Reverse transcription of RNA and quantitative real-time PCR was performed using the Hairpin-it^TM^ miRNAs qPCR Quantitation Assay Kit (GenePharma) according to the manufacturer's instructions. Quantitative RT-PCR was performed in a Roche 480 real-time PCR system. The 2^−ΔΔ*Ct*^ method was used to evaluate the miR-622 gene expression after normalization for expression of the endogenous controls U6 (U6 non-coding small nuclear RNA). All primers for miR-622 and the U6 genes were synthesized and approved by GenePharma. Each experiment was repeated at least three times.

#### Western Blotting

Total proteins were extracted with RIPA lysis buffer (Beyotime) with Protease Inhibitor Cocktail (Roche) and Phosphatase Inhibitor Cocktail (Roche). Protein samples were separated in sodium dodecyl sulfate (SDS)-PAGE and transferred to polyvinylidene fluoride (PVDF) filter membranes (Millipore, USA). After blocking in phosphate buffered saline (PBS) containing 0.05% Tween-20 and 5% non-fat milk powder, the membranes were incubated with the following primary antibodies: RNF8 (Santa Cruz Biotech, 1:500), EMT kit (Cell Signaling Technology, 1:2000), beta-actin (Santa Cruz Biotech, 1:4000), Secondary antibodies were Horseradish peroxidase (HRP)-conjugated anti-mouse IgG (ZB-2305, ZSGB-Bio, 1:4000) or anti-Rabbit IgG(Fc) (ZB-2301, ZSGB-Bio, 1:4000). Subsequent visualization was detected by Digit imaging system (Thermo, Japan), the gray level of the bands was quantitated by ImageJ software.

#### *In vitro* Cell Viability Assays

To perform cell viability assays, cells were counted and plated in the well of 96-well plate (1,500 cells per well) 24 h after transfection of chemically modified oligonucleotides or siRNA. the cell viability ability was determined using the Cell Counting Kit-8(CCK8) Assay Kit (Dojindo Corp, Japan) according to the manufacturer's protocol: After the 0/24/48/72 h proliferation of cells, the kit reagent dissolved with RPIM1640 Medium to prepare a 10% working reagent. The original medium was removed and 110 ul working reagent was added to each well. After 2 h incubation in the 37°C incubator. The absorbance was measured at 450 nm to calculate the number of cells, the cell viability assays were performed three independent times.

#### *In vitro* Transwell Migration Assays

Cell migration assays were performed using a 24-well plate with 8-μm Transwell Chambers (Costar). Twenty-four hours after transfection of synthesized miRNA oligonucleotide or siRNA, cells were digested by 0.25% Trypsin (Hyclone) and resuspended with non-serum culture medium (DMEM). After cell counting, 5 × 10^4^ cells were suspended in 300 μL DMEM and seeded into the upper well of Transwell Chamber. The well below the Transwell Chamber, 600 μL of DMEM supplemented with 10% fetal bovine serum was added to stimulate the migration of cells. After incubation for 24 h at 37°C and 5% CO_2_, the Transwell chambers were removed from the 24-well plate, and non-migrated cells were removed from the upper surface of the membrane by cotton swabs. Cells that moved to the bottom surface of the chamber were fixed with 5% paraformaldehyde (Sinopharm Chemical Reagent Co., Ltd) for 10 min and stained with Hematoxylin-Eosin (HE) staining method. Then, the membrane with cells was imaged and counted in at least five random fields. The assay was performed three independent times.

#### Statistical Analysis

Statistical analysis was conducted using the SPSS statistical software program (Version 13.0; SPSS Inc.). All results were presented as the mean ± standard error of the mean (SEM). Student *t*-test and One-way ANOVA was performed to compare the differences between treated groups relative to their paired controls. *p*-values are indicated in the text and figures above the two groups compared and *p* < 0.05 (denoted by asterisks) was considered as statistically significant.

## Results

### *In silico* Exploration of miR-622 Targets and Their Prognostic Values

To evaluate the prognostic values of miR-622 in various cancers, we performed a pan-cancer survival analysis based on the pan-cancer TCGA miRNA database (39). The results showed that the patient survival rate was negatively correlated with high levels of miR-622 in breast cancer (BC, *p* = 2 × 10^−6^), cervical squamous cell carcinoma (CISCC, *p* = 0.0195), head and neck squamous cell carcinoma (HNSC, *p* = 0.0011), ovarian cancer (OV, *p* = 1.3 × 10^−5^), pancreatic ductal adenocarcinoma (PDAC, *p* = 0.0018), and uterine corpus endometrial carcinoma (UCEC, *p* = 4.5 × 10^−4^) (Supplementary Figure 1). However, esophageal adenocarcinoma (EA), esophageal squamous cell carcinoma (ESCC), kidney renal papillary cell carcinoma (KIRP), lung adenocarcinoma (LUAD), pheochromocytoma and paraganglioma (PCPG), rectum adenocarcinoma (READ), sarcoma (SARC), testicular germ cell tumor (TGCT), and thymoma (THYM) were not correlated with miR-622 abundance (data not shown). Interestingly, high miR-622 abundance was positively correlated with the overall patient survival rate in bladder carcinoma (BCa, *p* = 0.017), kidney renal clear cell carcinoma (KIRC, *p* = 7.3 × 10^−11^), liver hepatocellular carcinoma (LIHC, *p* = 2.3 × 10^−10^), lung squamous cell carcinoma (LUSC, *p* = 0.0035), stomach adenocarcinoma (STAD, *p* = 0.018), and thyroid carcinoma (THCA, *p* = 0.0037), which indicated that miR-622 might play distinct roles in different cancers. These pan-cancer survival results also demonstrated that miR-622 could be a prognostic marker for many cancers, especially BC, OV, UCEC, KIRC, and LIHC (Supplementary Figure 1).

**Figure 2:**
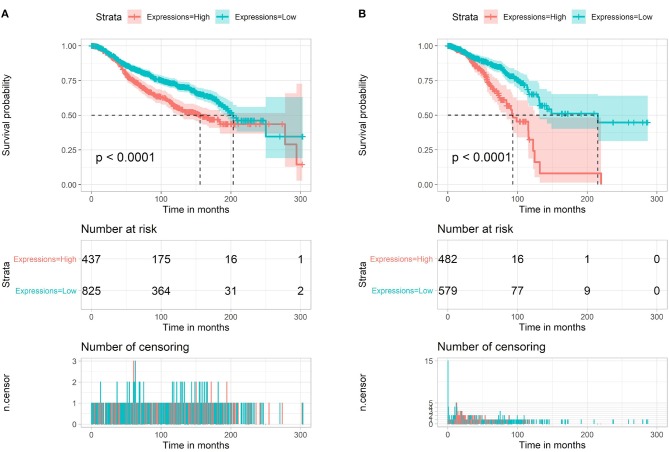
Survival analysis of miR-622. Overall survival (OS) of miR-622 based on METABRIC (1,262 breast cancer samples, **A**) and TCGA (1,077 breast cancer samples, **B**).

In breast cancer, we also analyzed the miR-622 survival rate in the METABRIC database (1,262 breast cancer samples). We found that high miR-622 abundance was significantly correlated with a poor prognosis in the METABRIC (Figure 2A) and TCGA (Figure 2B) datasets. In summary, these analyses indicated that miR-622 might be a potential marker for the prognosis of breast cancer patients.

**Figure 3:**
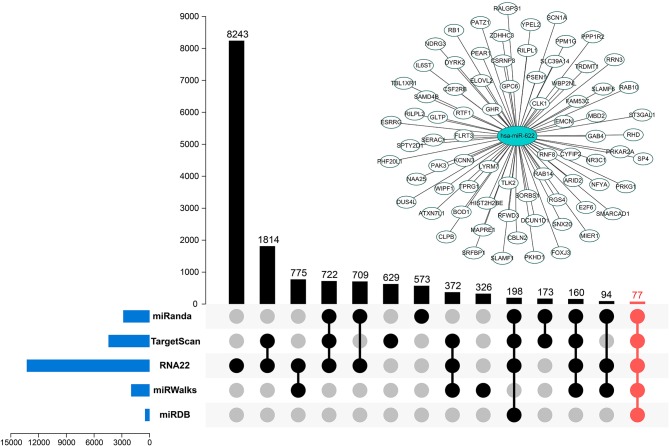
Potential targets predicted by multiple tools. Five promising miRNA-targets prediction tools including miRanda, TargetScan7.2, RNA22 v2.0, miRWalks v3.0, and miRDB were used to find the targets of miR-622. The intersection of five tool's results was marked in red. miR-622-targets interaction network was visualized using Cytoscape 3.7.1.

To understand how miR-622 participates in different biological processes, based on the regulatory patterns of miRNAs, the potential targets of miR-622 were identified with miRWalk 3.0 and 4 other highly recognizable and promising miRNA target prediction tools. Each prediction tool gives a set of predicted target genes. The results of the intersection of these five predicted target gene sets were integrated and visualized with TBtools software and the R package UpSetR (Figure 3) (31, 32). Finally, 77 overlapping genes, including YPEL2, RNF8, and RB1, were both predicted by the five tools (Figure 3 and Supplementary Table 1), indicating that these genes could be promising targets of miR-622. On the other hand, these genes might be involved in miR-622-regulated biological processes.

### Bioinformatics Analysis of the Predicted Target Genes

#### GO Annotation and KEGG Enrichment Analyses

**Figure 4:**
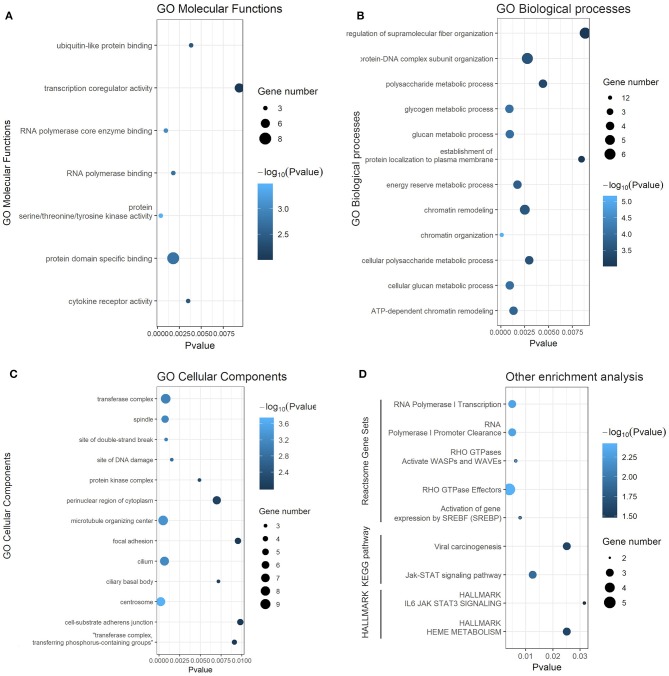
Gene ontology and KEGG pathway enrichment analysis of predicted targets of miR-622. Gene ontology (GO) annotation analysis and KEGG pathway enrichment analysis results. Each bubble represents a term, and its size represent the counts of involved genes. Lighter colors indicate smaller *P*-values. **(A)** Enriched terms of GO molecular functions (MFs, *p* < 0.01). **(B)** Enriched terms of GO biological process (BPs, *p* < 0.01). **(C)** Enriched terms of GO cellular compounds (CCs, *p* < 0.01). **(D)** Enriched terms of Reactome, KEGG pathway, and HELLMARK (*p* < 0.05).

To obtain a better understanding of the function and regulatory pattern of miR-622 at the cellular level, analyses including GO annotation and KEGG pathway enrichment of the 77 overlapping target genes of miR-622 were performed using the web-based functional enrichment tool Metascape (33) and KOBAS (40, 41). Terms with *P*-values <0.05 (KEGG and HALLMARK) or *P* values <0.01 (GO) were visualized with R language as a bubble plot. As shown in the results, terms such as ubiquitin-like protein binding and RNA polymerase core enzyme binding were enriched in GO molecular functions (MFs) (Figure 4A). Regarding GO biological processes (BPs), these 77 targets were mainly enriched in chromatin organization, remodeling, the glucan metabolic process, the protein-DNA complex subunit and so on (Figure 4B). Regarding cellular components (CCs), the target genes were commonly enriched in the centrosome, microtubule organizing center, transferase complex, and site of DNA damage (Figure 4C). Furthermore, the Jak-STAT signaling pathway and its hallmarks were both enriched by KEGG pathway enrichment and HALLMARK enrichment. KEGG enrichment and HALLMARK enrichment, as well as viral carcinogenesis and HEME metabolism, were also significantly enriched (Figure 4D). To further explore the correlation between miR-622 and disease, KEGG DISEASE, and GWAS Catalog were also examined with KOBAS using the 77 overlapping genes, and the results showed that cancers such as breast cancer, glioma, and bladder cancer were enriched (Supplementary Figure 2). Interestingly, breast size was also included in the GWAS catalog. These results indicate that there might be a potential relationship between breast cancer and miR-622.

In addition, to avoid the lack of key information that results from merely taking the intersection of the predicted targets into consideration, a functional enrichment analysis was also performed on all predicted target genes with the newest version of miRWalk, which contains the newest version of the search module of the pathway gene sets (30); 3,039 target genes with a miRWalk score >0.95 were all used as input for the functional enrichment analysis. The results showed that compared with the enrichment results of the 77 genes, the MF, CC, and BP GO terms were somewhat different. Regarding MFs, ubiquitin-related terms such as ubiquitin protein ligase binding, activity, ubiquitin-conjugating enzyme binding and K63-linked polyubiquitin binding were significantly enriched (Supplementary Figure 3B), indicating a close association between miR-622 and the ubiquitination process. Regarding GO BPs, DNA repair-related and ubiquitin-related terms were enriched (Supplementary Figure 3C). Regarding GO CCs, the ciliary tip, base, neuronal cell body membrane and synaptic vesicle membrane were significantly enriched (Supplementary Figure 3D). In the KEGG pathway enrichment analysis, terms such as pathway in cancer and MAPK signaling pathway were enriched, indicating that miR-622 might be involved in carcinogenesis and MAPK1-related cell proliferation.

#### Construction and Clustering of the PPI Network

**Figure 5:**
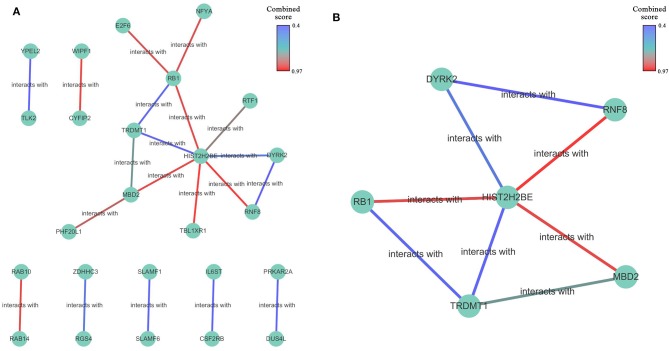
The protein-protein interaction network of overlapping genes. **(A)** Network was constructed by STRING database for overlapping 77 genes predicted by five promising miRNA-targets prediction tools with a medium confidence (interaction score >0.400). disconnected nodes were also removed. The network was visualized by Cytoscape 3.7.1 software. **(B)** Built-in app MCODE was utilized to find clusters in whole network.

A protein-protein interaction network based on 77 overlapping genes was constructed by the STRING database (Supplementary Figure 4) and can be visualized in Figure 5A. The results showed that, with the exception of disconnected nodes, 25 genes were connected to another, with 11 genes in one network. These 25 genes were selected as hub genes, which might play important roles in the miR-622-related regulation of cellular processes. By comparing these 25 genes with those that were differentially expressed in invasive breast cancer (BRCA), we found that three genes, HIST2H2BE, RGS4, and RAB10, were included in the intersection (Supplementary Figure 5); therefore, the miRNA-target axis formed by miR-622 and these genes might be involved in BRCA. To find the potential interconnected regions in this network, MCODE was utilized. A smaller network with 6 nodes (RNF8, RB1, DYRK2, HIST2H2BE, TRDMT1, and MBD2) and 8 edges were clustered (Figure 5B). These genes might be pivotal genes involved in miR-622-regulated biological processes, which could be further selected for experimental validation.

#### Pan-Cancer Expression Analysis of the Hub Genes

To understand the roles of these 25 hub genes in various cancers, we analyzed the expression patterns of these hub genes in cancer and normal tissues in the TCGA database. The result is shown in Supplementary Figure 6, which shows an expression matrix heatmap based on the given list of hub genes. The heatmap showed that these hub genes exhibited distinct expression patterns in different cancers. A specific gene with highly differential expression, such as RNF8 in THYM, YPEL2 in LAML, and E2F6 in DLBC, indicates a strong correlation between this gene and the corresponding cancer.

#### Prognostic Potential of the Hub Genes

**Figure 6:**
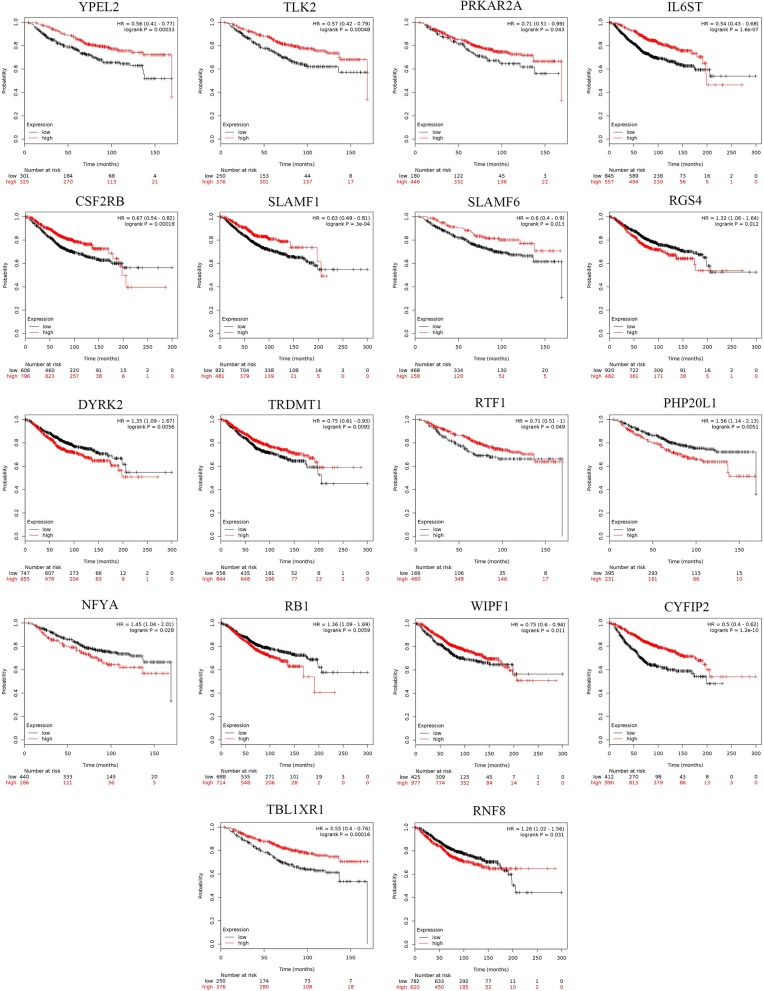
The survival analysis of 25 hub targets in breast cancer and pan-cancer survival heatmap. The overall survival (OS) rate of 25 hub targets by Kaplan-Meier survival analysis based on 1,402 samples downloaded from Kaplan-Meier plotter tool.

The prognostic values of the 25 hub genes selected by the PPI network in breast cancer were determined by Kaplan-Meier plotter (http://kmplot.com/analysis/) and GEPIA, to acquire a promising judgement of whether these hub genes can be used as prognostic markers. During the analysis, Jetset probes were used as the proper probe for the promising expression of specific genes (37). The results showed that in BRCA, the high expression of RGS4, DYRK2, PHP20L1, NFYA, RB1, RNF8 (Figure 6) was negatively correlated with patient survival, while YPEL2, TLK2, PRKAR2A, IL6ST, CSF2RB, SLAMF1, SLAMF6, TRDMT1, RTF1, WIPF1, CYFIP2, and TBL1XR1 (Figure 6) exhibited the opposite correlation: the high expression of these hub genes indicates a better prognosis. These genes can be used as potential prognostic markers for breast cancer. Furthermore, the survival rate heatmap including OS (Supplementary Figure 7A) and RFS (Supplementary Figure 7B) was also plotted based on the TCGA BRCA dataset, which might also be a reference for prognostic markers' selection in various cancers.

#### Predicted Values of the Hub Genes

A predictive marker predicts the benefits from a specific treatment and can help select a particular treatment over another. To explore the predictive potentials of these hub genes in breast cancer, ROC plotter, the first online transcriptome-level validation tool for predictive biomarkers, was utilized to find the potential predicted values of these genes (37, 38). The results showed that HIST2B, DYRK2, and RB1 might be used as predictive markers for breast cancer (Supplementary Figure 8).

### Validation of the miR-622-RNF8 Axis

#### miR-622 Inhibits RNF8 Expression via Direct Targeting of the RNF8 3'-UTR

**Figure 7:**
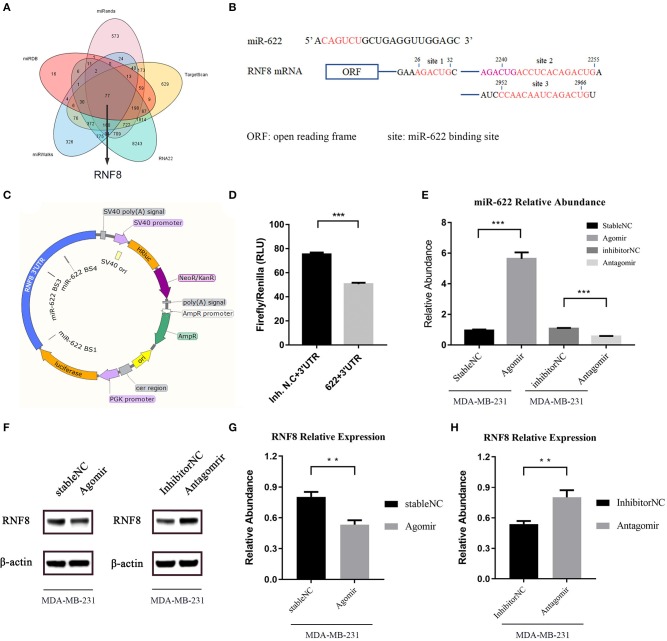
miR-622 inhibits RNF8 expression. **(A)** The prediction of miR-622-targeting mRNAs by five online tools. There are 77 genes in the intersection of five algorithms' results set. **(B)** Sequences of the predicted miR-622 binding sites of the RNF8 mRNA 3'UTR, with binding areas marked in red or pink (if two sites share the same sequence). **(C)** Structure of dual-luciferase plasmid inserted with RNF8 3'UTR. **(D)** Forty 8 h after the liposome-mediated transient transfection of miR-622 agomir/stable NC/antagomir/inhibitor NC and dual-luciferase plasmid, cells were lysed and dual-luciferase assay was performed to detect the fluorescence of firefly luciferase and renilla luciferase. Relative Light unit (RLU) was obtained by the ratio of Firefly fluorescence and Renilla fluorescence. **(E)** Real-time PCR was performed to detect the abundance of miR-622 in MDA-MB-231 cells treated with agomir/antagomir **(F–H)**. Western blot assay was performed to measure the expression of RNF8 in treated cells, results was obtained by gray level analysis of the band of western blot. miR-622 RNA levels are expressed as the mean ± SEM of four different experiments normalized to U6 abundance. The RNF8 protein levels are expressed as the mean ± SEM of three different experiments normalized to β-actin levels. ^**^p < 0.01, ^***^p < 0.001 vs. control.

In the bioinformatic target prediction of miR-622, 77 genes were identified in the intersection of the predicted target sets from five tools showing relatively high credibility for experimental validation (Figure 7A). Through a literature investigation, we found that RNF8, a predicted target hub gene that was also selected in the PPI network, was recently proven to promote EMT process and therefore facilitate breast cancer metastasis (12–14). In addition, reverse miRNA prediction using the RNF8 3'-UTR showed that miR-622 is one of 7 miRNAs predicted by all five tools used previously (Supplementary Figure 9), further suggesting that miR-622 might regulate RNF8.

In the functional enrichment analysis, miR-622 was mainly involved in ubiquitin-related compounds and pathways, such as K63-linked polyubiquitin binding, ubiquitin protein ligase activity, and ubiquitin-like protein binding (Figure 4A and Supplementary Figures 3B–D), and DNA damage repair-related compounds and pathways, such as sites of double-strand breaks, sites of DNA damage, and the positive regulation of DNA repair (Figure 4C and Supplementary Figures 3A,C). Interestingly, to our knowledge, these are both functions of RNF8. RNF8 is an E3 ubiquitin protein ligase, and its common biological function is to catalyze the ubiquitination of target proteins, inducing the degradation of proteins or participating in the DNA damage response (DDR). In summary, these results, combined with our prediction and functional enrichment analyses of miR-622, suggesting that RNF8 and miR-622 could be functionally connected, and that might result from the regulation of miR-622 on RNF8.

Two recent articles reported that RNF8 could induce the K63-linked ubiquitination of the transcription factor Twist and promote EMT in breast cancer cells, which leads to breast cancer metastasis (12, 13). These results revealed that RNF8 was a pivotal molecule in breast cancer cell EMT process. Since RNF8 might be a crucial target for miR-622, we hypothesized that miR-622 might regulate the EMT process via the direct regulation of RNF8. Additionally, the relationship between miR-622 and breast cancer has not yet been studied, but we found a potential correlation between miR-622 and breast cancer (Supplementary Figures 2B,D). We therefore chose the miR-622-RNF8 axis in breast cancer as the *in vitro* proof of concept for our bioinformatic target analysis of miR-622.

To validate the regulation of RNF8 by miR-622 *in vitro*, the potential binding sites of miR-622 and the RNF8 3'-UTR were screened and are shown in Figure 7B. Then, the full-length 3'-UTR sequence of RNF8 was cloned and linked behind the 3' end of the coding sequence of a luciferase to simulate the natural transcriptional inhibition of miR-622 on RNF8. A dual-luciferase system was also introduced to avoid the difference in liposome-mediated transfection (Figure 7C). Forty-eight hours after cotransfection of the dual-luciferase plasmid (pmirGLO-RNF8) and miR-622 mimics (agomir), the expression of luciferase was decreased significantly compared to that of the control group (Figure 7D). This result demonstrated that miR-622 can directly target the RNF8 3'-UTR and regulate upstream luciferase expression. To further support our hypothesis that miR-622 inhibited the expression of RNF8 *in vitro*, MDA-MB-231 breast cancer cells (a cell line with high expression of RNF8) (12) were transfected with the miR-622 agomir and antagomir, respectively. The results showed that miR-622 abundance in MDA-MB-231 cells was vastly upregulated (5.8-fold, Figure 7E *p* < 0.001), and the RNF8 protein level was significantly downregulated accordingly (Figures 7F,G, *p* < 0.01). In contrast, when endogenous miR-622 was downregulated by transient transfection of the chemically modified miR-622 antagomir (downmodulated ~0.5-fold, *p* < 0.001, compared with the inhibitor (NC), Figure 7E, right), the RNF8 protein level was significantly increased (~1.6-fold compared to the inhibitor N.C., Figure 7H, *p* < 0.01). Combined with the results of the dual-luciferase assay showing that miR-622 could bind to RNF8 at its 3'-UTR to induce posttranscriptional silencing, we conclude that miR-622 can directly regulate RNF8 in breast cancer cells, verifying the miR-622-RNF8 axis.

#### miR-622 Inhibits the Epithelial-Mesenchymal Transition of Breast Cancer Cells and Affects the Cell Viability and Migration Capacity of Breast Cancer Cells

**Figure 8:**
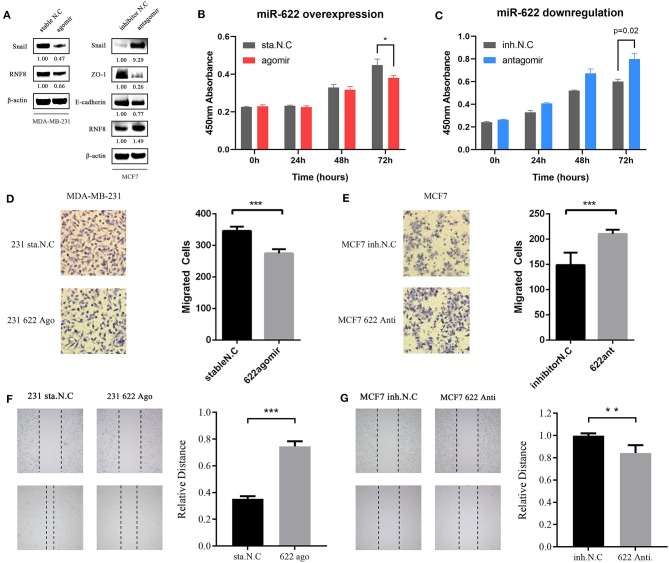
miR-622 inhibits breast cancer cell EMT, cell viability and migration via RNF8. **(A)** MDA-MB231 and MCF7 cell was transfected with miR-622 agomir and antagomir respectively. Thirty-six hours after transfection, cells were harvested and lysed to extract total proteins, then western blot assay was performed to detect changes in EMT-related hallmarks (Epithelial status hallmarks: E-cadherin, ZO-1; Mesenchymal status hallmarks: Snail). **(B,C)** a CCK8 cell vitality assay was performed to detect cell viability capacity of miR-622-overexpressing MDA-MB-231 cell **(B)** and miR-622-knockd MCF7 cell **(C)**. **(D,E)** Representative microscopic images and cell counts of migratory cells from the miR-622-mimic (agomir)-transfected MDA-MB-231 breast cancer cell group **(D)** and the miR-622-inhibitor (antagomir)-transfected MCF7 breast cancer cell group **(E)** in a Transwell assay; **(F,G)** Representative microscopic images and a relative distance of wound healing assay in miR-622 overexpressing MDA-MB-231 cells **(E)** and miR-622 knockdown MCF7 cells **(F)**. Data from the CCK8 cell viability assay, Transwell migration assay and wound healing assay represent the mean ± SEM of three independently prepared samples. ^*^p < 0.05, ^**^p < 0.01, ^***^p < 0.001 vs. control.

Because RNF8 can induce the EMT process in breast cancer and miR-622 can directly regulate RNF8 expression, we hypothesized that miR-622 can regulate the EMT process via the regulation of RNF8. Epithelial-mesenchymal transition can be characterized via molecular level alterations in EMT-related signatures, including epithelial hallmarks such as E-cadherin, ZO-1, and Claudin-1, and mesenchymal hallmarks, such as Vimentin, N-cadherin, and Snail. To explore whether miR-622 can regulate EMT via RNF8, we upregulated miR-622 in MDA-MB-231 cells and downregulated miR-622 in MCF7 cells and then collected the cells for the western blot detection of EMT-related hallmarks. The results showed that the overexpression of miR-622 could downregulate the expression of RNF8 and Snail, a mesenchymal hallmark, indicating that miR-622 inhibited the EMT process in breast cancer cells. In addition, the cells were transfected with the miR-622 antagomir and the inhibitor N.C. in the breast cancer cell line MCF7 for Western blot detection. The results showed that the knockdown of miR-622 could upregulate the expression of RNF8 as well as the expression of the mesenchymal status marker Snail (by 0.65-fold, Figure 8A), while the epithelial status markers E-cadherin, ZO-1, and Claudin-1 were significantly decreased (by 0.65-fold, Figure 8A).

In the functional enrichment analysis, miR-622 was found to be involved in cell proliferation-related signaling pathways, such as the Jak-STAT pathway, the Ras pathway, and the MAPK1 pathway (Figure 4A and Supplementary Figures 3A,B). We therefore believe that miR-622 might be involved in the regulation of cell viability. In addition, after proving the regulation of RNF8 expression by miR-622, we believe it is reasonable to assume that the RNF8-induced increase in breast cancer cell migration capacity might be directly regulated by miR-622.

To explore the effect of miR-622 on the migration and viability potential of breast cancer cells, we overexpressed miR-622 in MDA-MB-231 cells via transfection of the miR-622 agomir and stable N.C. Thirty-six hours after transfection, RNF8 protein level alterations were first verified by western blot. Then, the CCK8 cell viability (Figures 8B,C), Transwell (Figures 8D,E), and wound healing assays (Figures 8F,G) were performed. The results showed that the number of migrated cells was decreased significantly in the miR-622 overexpression group compared with the control group transfected with stable N.C (Figure 8C, *p* < 0.001), indicating that the upregulation of miR-622 inhibits the migration capacity of breast cancer cells. The results were further supported by wound healing assays (Figure 8E, *p* < 0.001), which showed that upregulated miR-622 inhibited cell mobility. To further support our hypothesis, MCF7 cells were transfected with the designed antagomir to reduce the level of miR-622. Cell viability and migration assays were then performed. The results showed that decreased miR-622 promoted the viability (Figure 8B, *p* < 0.001) and migration (Figures 8D,F, *p* < 0.001 and *p* < 0.01, respectively) capacities of breast cancer cells. In summary, these results showed that miR-622 inhibited breast cancer cell migration and viability *in vitro*.

#### Rescue Experiments in Breast Cancer Cell MCF7 Showed That miR-622-Mediated Migration and EMT Depend on RNF8

**Figure 9:**
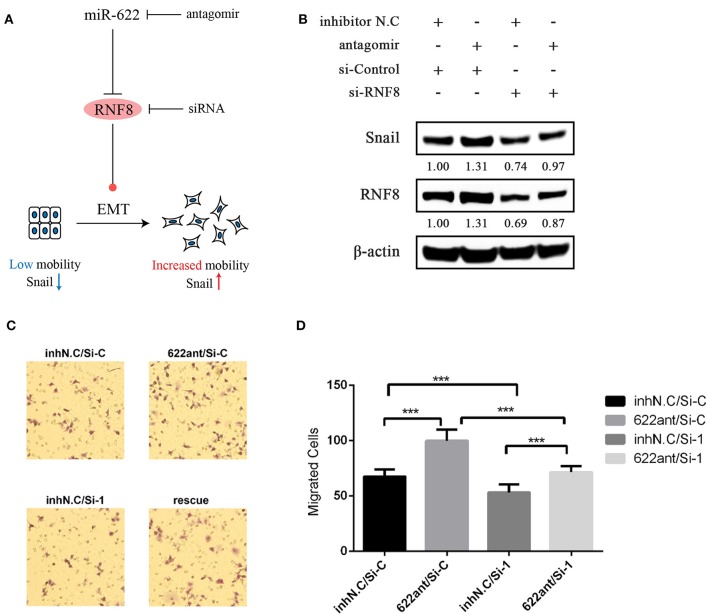
Rescue experiments demonstrating that RNF8 is a direct functional target of miR-622 in EMT and breast cancer cell migration. **(A)** Schematics of rescue experiment. MCF7 breast cancer cells were treated with the combination of miR-622 antagomir (Anti), its control inhibitor NC (Inh N.C), RNF8 siRNA (si-RNF8), and its control si-Control (si-C). **(B)** Western blot was then performed to detect the expression of RNF8 and the EMT hallmark protein Snail. **(C,D)** Transwell assays were performed to detect the migration capacity of treated cells. **(C)** Representative examples and **(D)** quantification of the Transwell assays. Data in Transwell migration assays represent the mean ± SEM of three independently prepared samples, each measured five times. ^***^p < 0.001 vs. control.

We verified the direct regulation of RNF8 by miR-622 and the relationship between miR-622 and the EMT process. To further test whether the miR-622-induced EMT process and EMT-related phenotype changes (for example, migration) of breast cancer cells are directly dependent on RNF8, an siRNA designed against RNF8 (siRNF8) and a miR-622-inhibitor (antagomir) were cotransfected to knock down the expression of RNF8 and miR-622 in breast cancer cell MCF7, respectively (Figure 9A). The expression changes in EMT-associated markers (Snail, a hallmark of the mesenchyme status; ZO-1, a hallmark of the epithelial status) and migration capacity were subsequently examined to determine whether the phenotypes caused by miR-622 downregulation could be reversed by its target's (RNF8) inhibition. The results showed that the protein levels of RNF8 and Snail were significantly increased in the miR-622 antagomir-treated group (Figure 9B), while cell migration was largely increased, as determined by the Transwell assay. By further cotransfecting the cells with both the miR-622 antagomir and the RNF8-targeting siRNA, the results showed that following the siRNA-mediated downregulation of RNF8, the expression of Snail was partially reversed (Figure 9B). A similar result was also observed in the Transwell assay, which showed partially rescued migration capacity (Figures 9C,D). Taken together, these results demonstrate that miR-622 could regulate EMT and EMT-related functional phenotypes by directly regulating RNF8 expression.

## Discussion

Unraveling the molecular mechanisms underlying the initiation, development and metastasis of breast cancer would promote diagnosis, treatment, and prognosis evaluations. High-throughput platforms, such as microarrays and RNA sequencing, have been developing rapidly in disease progression, which not only provides the basis for the discovery of new targets for the diagnosis, therapy, and prognosis of cancers (42) but also gives us an overall view of molecular alterations.

In the present study, we focused on miR-622, a non-coding microRNA located in the q31.3 arm of human chromosome 13 NC_000013.11 that is involved in the formation and progression of many common cancers, such as gastric cancer (25), lung cancer (27), liver cancer (28, 43), glioblastoma (44), colorectal cancer (43), and acute myeloid leukemia (AML), by acting as a tumor suppressor by targeting ING1, HIF-1α, MAP4K4, YAP1, and RB1. However, some studies have shown that miR-622 may act as a protooncogene in colorectal cancer by targeting DYRK2 and inhibiting the migration and invasion of colorectal cancer cells (45). Thus, whether miR-622 is oncogenic or anti-cancer cannot be generalized due to different tumor environments. Based on miRNA-related studies, it is clear that the promotion or suppression of cancer may depend on its key target genes in the cells owing to the transcriptional regulatory pattern of the miRNA on its target genes. Therefore, identifying pivotal target genes of a miRNA might indicate how this miRNA regulates various cell signaling pathways and cancer processes in a larger picture, such as at the transcriptome level.

In the present study, we utilized five promising miRNA target prediction tools, miRWalk 3.0 (30), RNA22 2.0 (46), miRanda (47, 48), miRDB (49), and TargetScan 7.2 (50), to explore the potential target genes of miR-622. We found 77 genes at the intersection of the prediction of the five tools, showing that they might be promising targets of miR-622. Interestingly, among the 77 overlapping genes, DYRK2, YAP1, and RB1 have been proven to be direct target genes of miR-622 by Wang et al. (45), Xu et al. (51), and Ma et al. (52), respectively, further proving our predictions. Then, we used these 77 overlapping genes to perform GO annotation and KEGG pathway analyses, which might show us the crucial regulatory pattern of miR-622. The results showed that GO annotation items such as chromatin organization, remodeling, the protein-DNA complex subunit, and sites of DNA damage were enriched. KEGG pathway enrichment analysis revealed that the Jak-STAT signaling pathway and viral carcinogenesis were the most significant pathways. To unearth more functions of miR-622 and avoid the bias caused by taking only 77 overlapping genes into account, KEGG DISEASE and GWAS Catalog were also examined with KOBAS (Supplementary Figure 2). In addition, all targets with good scores predicted by miRWalk were subjected to GO annotation and KEGG pathway enrichment analyses (Supplementary Figure 3). These results indicate the potential correlation between miR-622 and breast cancer.

Through construction of the PPI network with the STRING database, we identified 25 hub genes that had high degrees of confidence, indicating that all of them might play pivotal roles in miR-622-regulated pathways and phenotypes. Therefore, we performed a pan-cancer expression analysis to examine the differential expression of these genes. The results showed that these hub genes exhibited distinct expression patterns in different cancers, such as RNF8 in THYM, YPEL2 in LAML and E2F6 in DLBC, which indicated the potential relation of these genes with the corresponding cancer. A pan-cancer survival rate analysis was also performed to assess the prognostic values of these genes, and the results showed that these overlapping genes might be signatures in various cancers, such as RNF8 in KIRC, RB1 in OV and E2F6 in LIHC. In breast cancer, 17 genes, including RNF8, DYRK2, RB1, and TRDMT1, could be used as prognostic biomarkers of breast cancer, indicating that these genes, including RNF8, might have a strong relationship with breast cancer.

MCODE was utilized to further identify the potential interconnected clusters in the PPI network. Finally, six genes, including RNF8, RB1, DYRK2, TRDMT1, and MBD2, were identified and regarded as hub target genes. Then, the predictive values of these genes were assessed with ROC plotter, a database containing a sufficiently large breast cancer cohort with transcriptomic and clinical response data from the GEO. The results showed that the high expression of DYRK2 and RB1 and the low expression of HIST2H2BE might be potential biomarkers for a good chemotherapy response. Interestingly, the regulation of RB1 and DYRK2 by miR-622 was already demonstrated *in vitro* by Ma et al. (52) and Wang et al. (45), which supported our prediction. However, the roles of miR-622 and RNF8, MBD2, TRDMT1, and HIST2H2BE have still not been discussed, requiring further exploration.

In the functional analysis of miR-622 targets, we found that miR-622 is mainly involved in ubiquitin-related compounds and pathways (Figure 4A and Supplementary Figures 1C, 3B,D) and DNA damage repair-related compounds and pathways (Figure 4C and Supplementary Figures 3A,C). Interestingly, these are both classic functions of RNF8. The common function of RNF8, an E3 ubiquitin protein ligase and one of six predicted hub target genes selected by STRING and MCODE (53, 54), is to catalyze the ubiquitination of target proteins, inducing the degradation of proteins or participating in the DNA damage response (DDR). These studies, combined with our prediction that RNF8 is a promising target of miR-622, corroborated the pivotal roles of RNF8 in miR-622-related biological processes (Figure 3 and Supplementary Table 1). Recently, Kuang et al. (12) and Lee et al. (13) reported that RNF8 could promote EMT in breast cancer cells, leading to breast cancer metastasis (12, 13). These studies demonstrate that RNF8 is a crucial regulator in breast cancer EMT process. Since our results indicated that RNF8 might be a crucial target for miR-622, we also found that some functions of miR-622 targets are associated with breast cancer (Supplementary Figures 2B,D). We reasonably assume that miR-622 might regulate the EMT process of breast cancer cells via the direct regulation of RNF8. In addition, to our knowledge, the relationship between miR-622 and breast cancer has not yet been studied. We therefore chose the miR-622-RNF8 axis in breast cancer as the *in vitro* proof of concept for our bioinformatic target analysis of miR-622.

First, by two-way miRNA target prediction using miR-622 and RNF8, the results showed that miR-622 could target RNF8 by binding its 3'-UTR and vice versa (Figure 7A and Supplementary Figure 9). Thus, we hypothesize that miR-622 might regulate breast cancer cell EMT process and migration as a tumor suppressor gene via the downregulation of RNF8. To support our hypothesis, we cloned the full-length sequence of the RNF8 3'-UTR into a dual luciferase system and thus showed the binding of miR-622 and the RNF8 3'-UTR *in vitro*. Furthermore, we found that the overexpression of miR-622 in breast cancer cells can downregulate the expression of RNF8, while miR-622 knockdown by the antagomir could increase the protein level of RNF8, which verified the regulation of RNF8 by miR-622. Then, by detecting EMT-related molecular signatures, we found that miR-622 could regulate RNF8-induced EMT by regulating RNF8. However, it is surprising that the relationship between miR-622 targets and EMT were not enriched in any enrichment analyses, this might because that miR-622 may not regulated EMT-related genes (such as E-cadherin) directly.

In addition, rescue experiments showed that the downregulation of EMT markers (Snail, Figure 9B) and enhanced migratory ability (Figures 9C,D) by the miR-622 antagomir were significantly reversed by siRNF8. Notably, the RNF8 protein levels in the “reverse” group were still slightly lower than those in the control group. The main cause of these results may be the fact that the “reverse” efficiency might be influenced by multiple factors, such as the efficiency of the miR-622 antagomir and the RNF8 siRNA and/or the transcriptional inhibition efficiency of miR-622 on RNF8 mRNA. Additionally, based on the miRNA regulation patterns, miR-622 is not the only regulatory miRNA of RNF8, because one gene can be regulated by multiple miRNAs and vice versa. In summary, these results experimentally identified RNF8 as a new target of miR-622 in breast cancer, revealing a new role for miR-622 in breast cancer tumorigenesis and verified our targets and functional analysis of miR-622. Also, we suppose that breast cancer metastasis might be regulated by miR-622 via the regulation of RNF8, which requires further supports via *in vivo* experiments.

To conclude, we utilized an integrated miRNA-target-phenotype research model that started from the bioinformatic identification and analysis of the miRNA-target axis, followed by mining and functional enrichment validation of the identified key miRNA-target axis, and end with performing miRNA-target-phenotype validation *in vitro* as proof of concept. Our experiments revealed not only the hub target genes of miR-622 *in silico*, demonstrating the potential functions of miR-622, but also the key regulatory mechanism of miR-622 in breast cancer viability and migration *in vitro* for the first time.

## Data Availability Statement

Publicly available datasets were analyzed in this study. These data can be found here: https://cancergenome.nih.gov/, http://gepia.cancer-pku.cn/, http://kmplot.com/analysis/ and https://www.ncbi.nlm.nih.gov/geo/. The detailed information can be found in figure legends.

## Author Contributions

CL, LM, and LZ designed the experiments. CL performed bioinformatic analysis the experiments, miR-622-RNF8 *in vitro* functional validation and wrote the manuscript. LM performed and analyzed the *in vitro* data. CZ contributed to the construction of dual-luciferase vector. JK contributed to the rescue experiment. X-YQ contributed to the functional analysis of predicted targets. CL, LM, and LZ edited the manuscript. LZ contributed to funding acquisition. All authors read and approved the final manuscript.

### Conflict of Interest

The authors declare that the research was conducted in the absence of any commercial or financial relationships that could be construed as a potential conflict of interest.

## Abbreviations

MiRNAs: microRNAs
GO: Gene oncology
KEGG: Kyoto Encyclopedia of Genes and Genomes
TCGA: The cancer genome atlas project
GEO: Gene Expression Omnibus
BC: Breast cancer
EMT: Epithelial-mesenchymal transition
3' UTR: 3'-untranslated regions
STRING: the Search Tool for the Retrieval of Interacting Genes
PPI: Protein-protein interaction
GEPIA: Gene Expression Profiling Interactive Analysis
GTEx: Genotype-Tissue Expression
ROC: Receiver operator characteristic curve
MFs: molecular functions
BPs: biological process
CCs: cellular compounds.
