# Supplementary material for: Bioinformatic Identification of miR-622 Key Target Genes and Experimental Validation of the miR-622-RNF8 Axis in Breast Cancer
Source: Front Oncol. 2019 Oct 23;9:1114. doi: 10.3389/fonc.2019.01114 (PMC6819436; doi:10.3389/fonc.2019.01114)
Supplement: Supplementary file 1 [file Data_Sheet_1.PDF]

Supplementary Material

1. Supplementary Figures and Tables

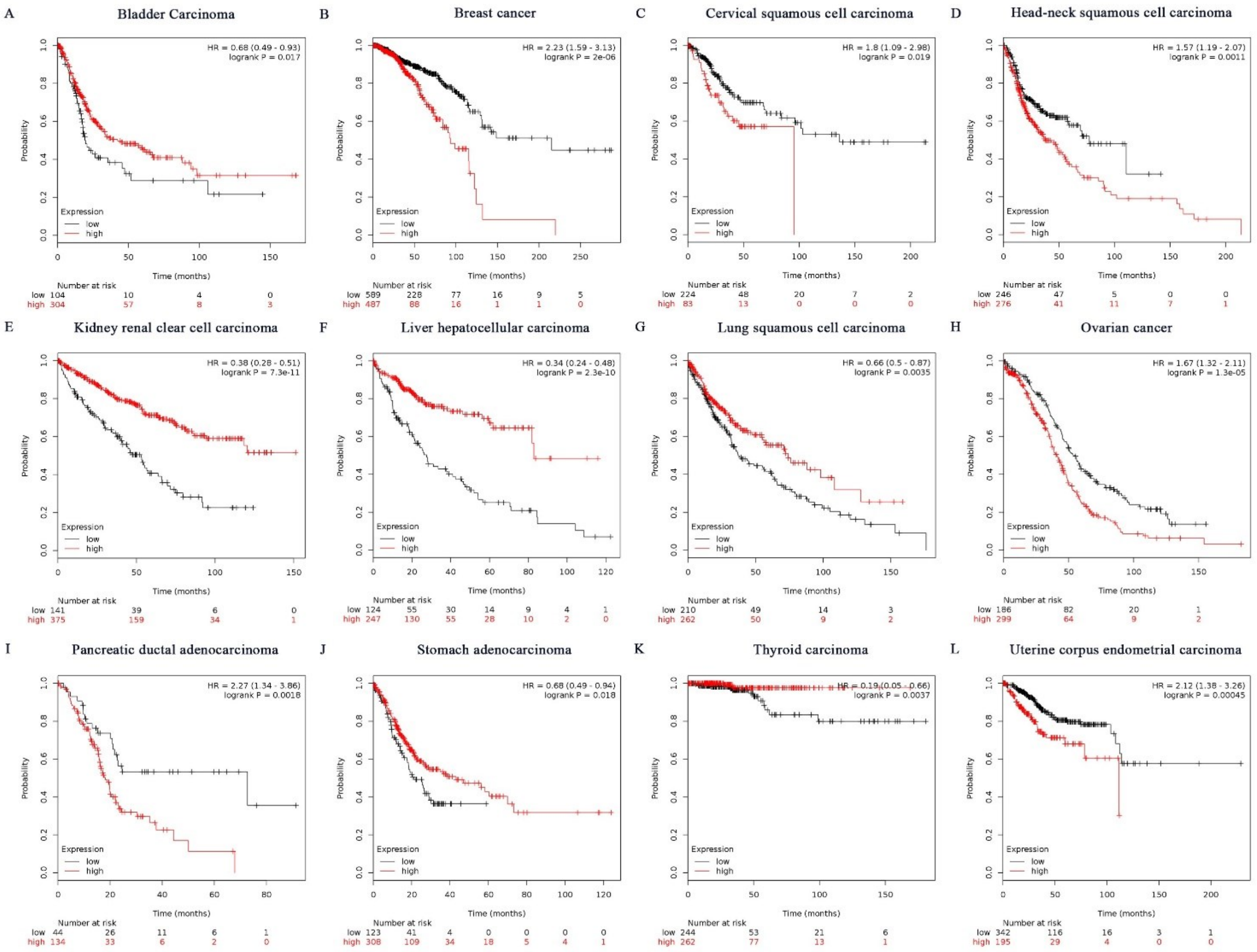

Supplementary Figure 1. The survival analysis of miR-622 in breast cancer based on TCGA database. (A-L) The overall survival (OS) rate of miR-622 by Kaplan-Meier survival analysis based on various TCGA cancers samples using Kaplan-Meier plotter tool. Only logrank P<0.05 were considered as significant and shown in the figure.

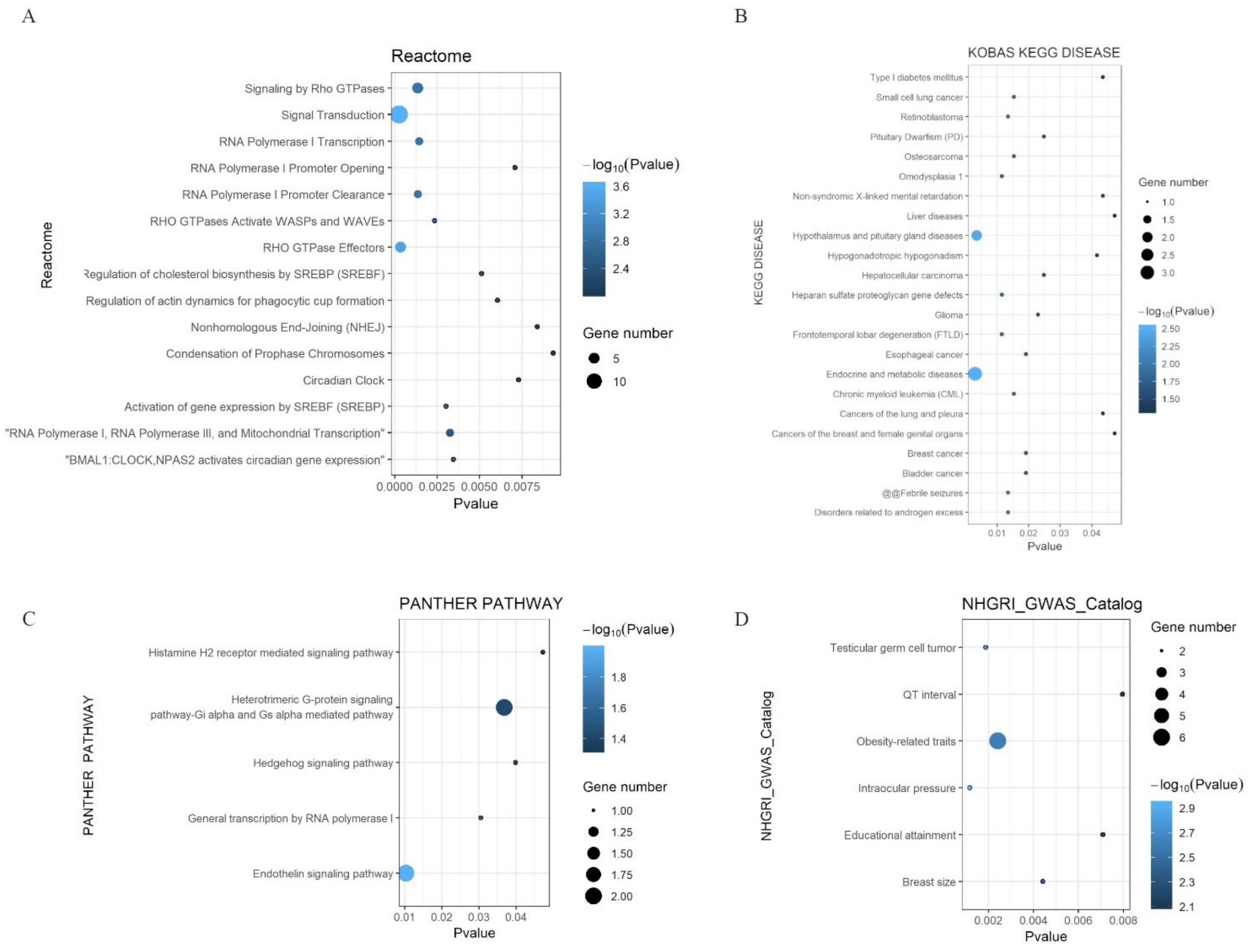

Supplementary Figure 2. Reactome, KEGG disease, PATHER pathway and GWAS_Catalog enrichment analysis of 77 overlapping predicted targets of miR-622. the 77 predicted genes of miR-622 were chosen to perform Reactome, KEGG disease, PATHER pathway and GWAS_Catalog analysis. Each bubble represents a term, and its size reprensent the counts of involved genes. Lighter colors indicate smaller P values. (A) Reactome annotation of target genes (p<0.01). (B) enriched items for KEGG disease (p<0.05). (C) enriched items for PATHER pathway (p<0.05). (D) enriched items for NHGRI_GWAS_Catalog (p<0.01)[1,2].

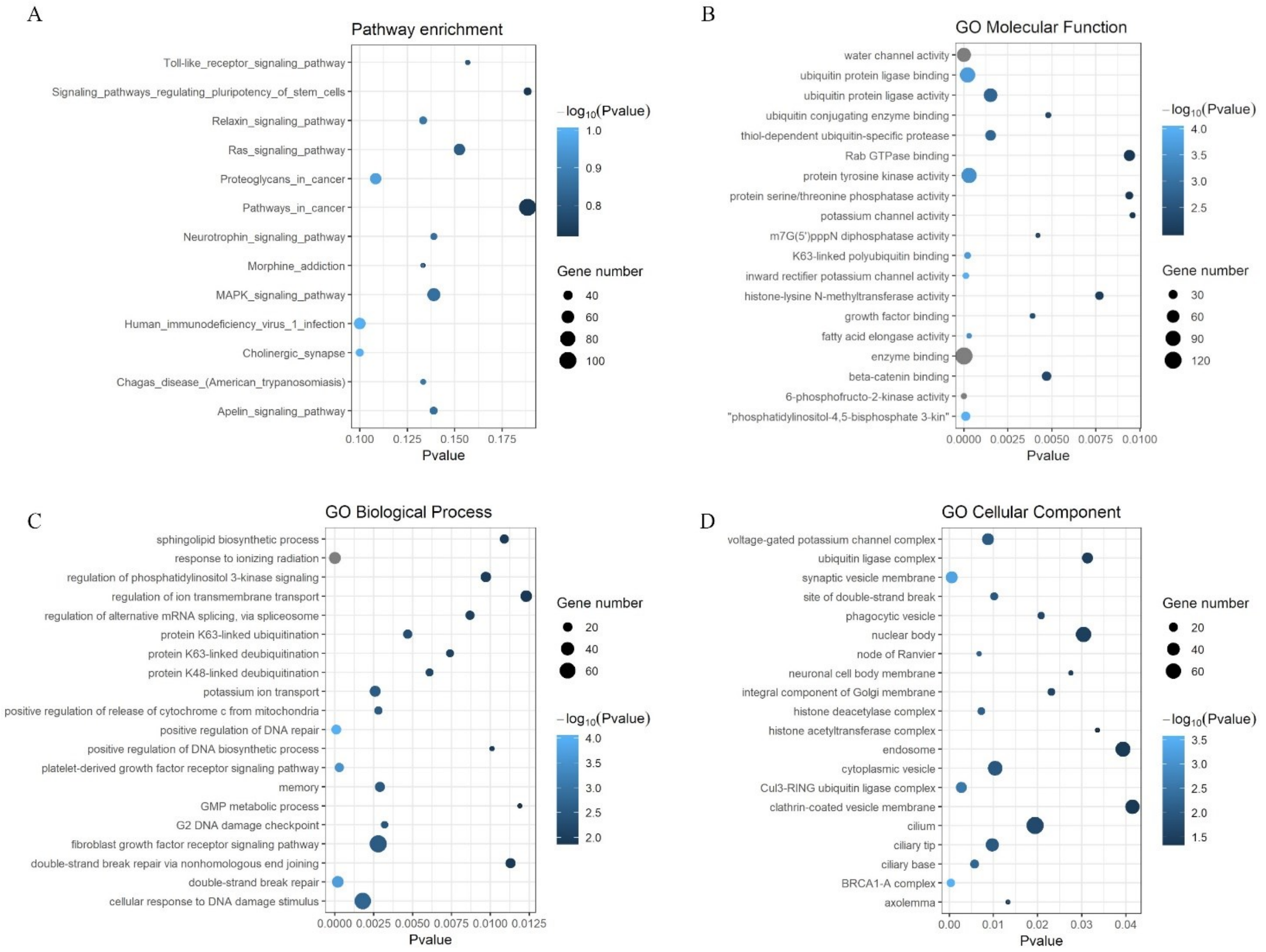

Supplementary Figure 3. Gene ontology and KEGG pathway enrichment analysis of predicted targets of miR-622 by miRWalks3.0. the predicted genes (score>0.95) of miR-622 by most new version 3.0 of miRWalks were chosen to perform GO annotation and KEGG pathway enrichment analysis results. Each bubble represents a term, and its size represent the counts of involved genes. Lighter colors indicate smaller P values. (A) Enriched terms of KEGG pathway. (B) Enriched terms of GO molecular functions. (C) Enriched terms of GO biological process. (D) Enriched terms of cellular compounds.

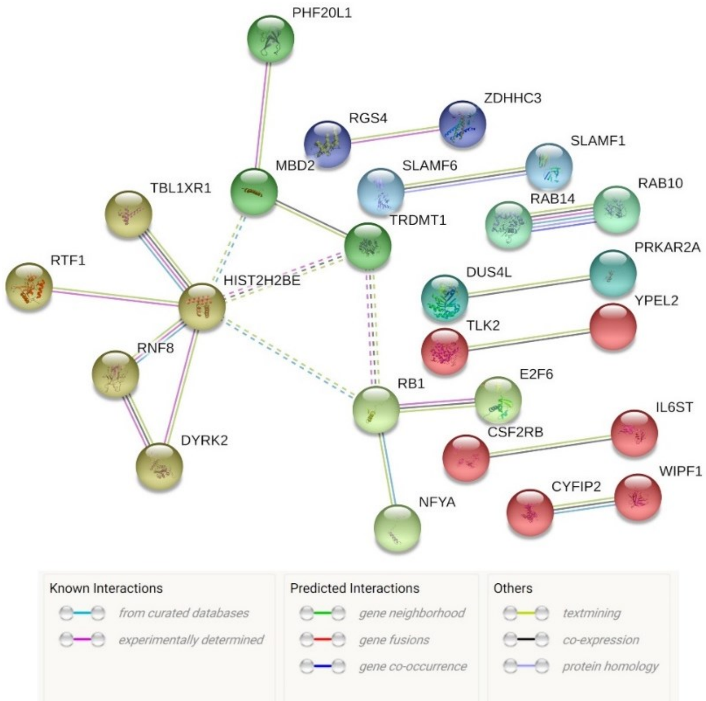

Supplementary Figure 4. The detailed protein-protein interaction network of overlapping genes. Protein-protein network was constructed by STRING database for overlapping 77 genes predicted by five promising miRNA-targets prediction tools with a medium confidence (interaction score>0.400). disconnected nodes were also removed.

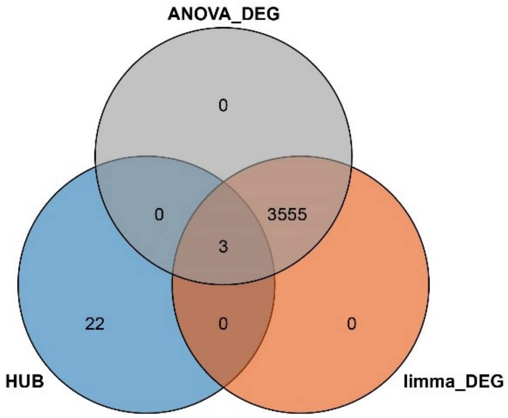

Supplementary Figure 5. The intersection of Differential expression genes (DEGs) in TCGA breast cancer and identified miR-622 hub genes. An online tools Gene Expression Profiling Interactive Analysis (GEPIA v2.0) were used to access TCGA dataset, "limma" (a package of R) and ANOVA method were used to find the DEGs in breast invasive carcinoma (BRCA) with a criterion that $\log_2|FC|>1$ and q-value<0.05. the unions and intersections of three subsets were visualized by TBtools. HIST2H2BE, RGS4, and RAB10 were in the intersection of three sets.

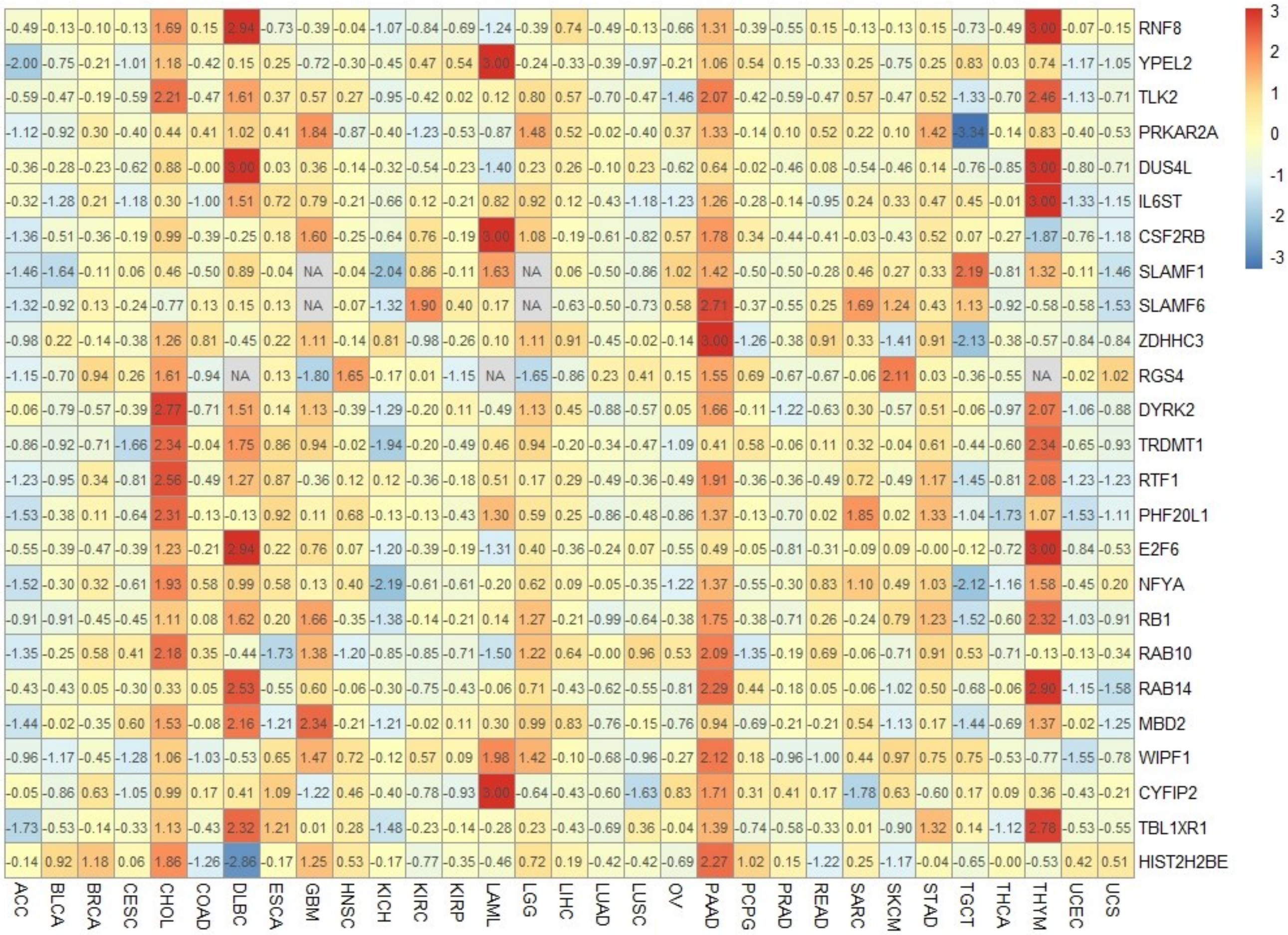

Supplementary Figure 6. The expression of 25 hub targets in different cancers. The expression of hub genes was based on TCGA samples in GEPIA and plotted by R package "pheatmap". The color in each block represents $\log_2|FC|$ value of a gene in tumor and corresponding normal tissue. Different genes in same tumors or normal tissues can be compared in one raw.

A

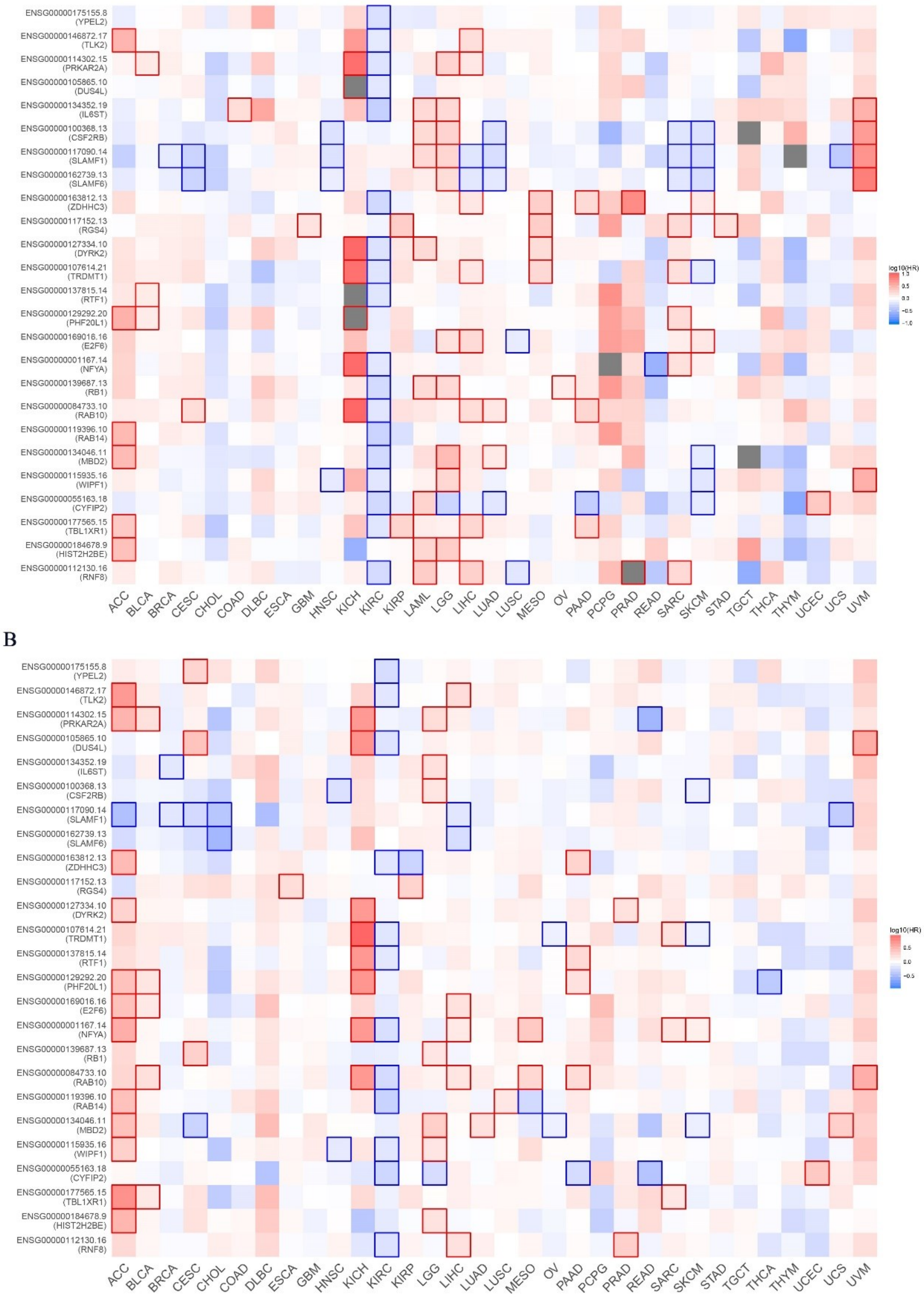

Supplementary Figure 7. The pan-cancer survival analysis heatmap of 25 hub gene. The heatmap of pan-cancer overall survival rate (A) and relapse-free survival rate (B) of **22 hub targets** by Kaplan-Meier survival analysis based on 1402 TCGA samples by GEPIA. A P-value<0.05 was considered to indicate a statistically significant difference.

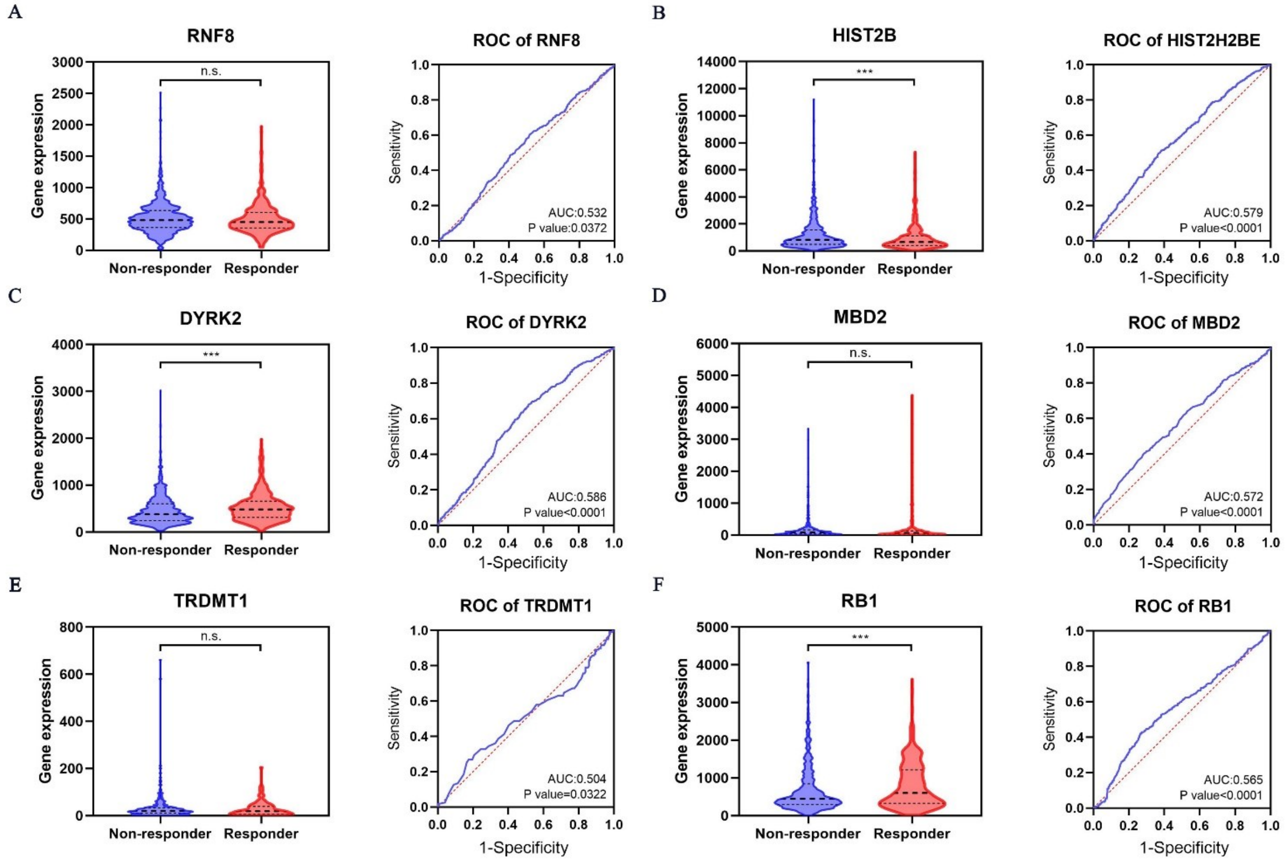

Supplementary Figure 8. The predicted potential of 6 hub targets in breast cancer. ROC plotter platform was used to check the predicted potential of 6 hub genes selected by MCODE module[3]. Each gene expression was compared between non-responding or responding group to any kind of chemotherapy including Taxane, Anthracycline, lxabepilone, CMF, FAC and FEC. Receiver operating characteristic curve (ROC) were plotted to measure the predictive potential of six hub genes

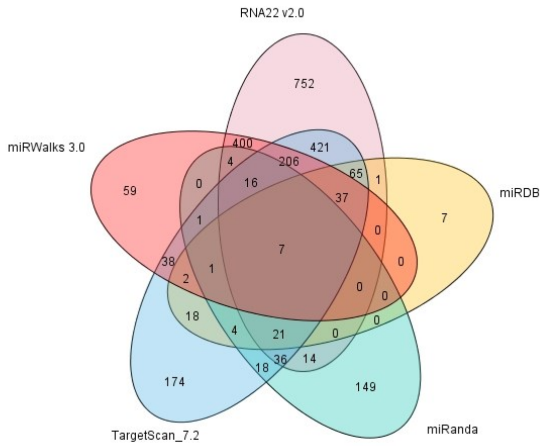

Supplementary Figure 9. The miRNAs prediction based on RNF8 3'untranslated region (3'UTR). The prediction of miRNAs targeting RNF8 mRNA 3'UTR. There are 7 miRNAs in the intersection of five algorithms' results set. Containing hsa-miR-4314, hsa-miR-622, hsa-miR-558, hsa-miR-1276, hsa-miR-3202, hsa-miR-761 and hsa-miR-4283.

Gene name	Gene name	Gene name	Gene name	Gene name	Gene name	Gene name	Gene name
PEAR1	GLTP	SLAMF1	SLC39A14	MIER1	RHD	FOXJ3	SNX20
IL6ST	RAB14	**RNF8**	YPEL2	ELOVL2	CYFIP2	PPM1G	BOD1
CLK1	RAB10	CSRNP3	SORBS1	SAMD4B	E2F6	RFWD3	ATXN7L1
PRKG1	EMCN	RGS4	WIPF1	DCUN1D1	PAK3	MAPRE1	SRFBP1
GAB4	SCN1A	RRN3	FLRT3	CBLN2	GPC6	SMARCAD1	KCNN3
FAM53C	RALGPS1	GHR	SPTY2D1	WBP2NL	SERAC1	NR3C1	SP4
PATZ1	RILPL1	ZDHHC3	PPP1R2	PKHD1	RTF1	NFYA	DUS4L
TLK2	RILPL2	MBD2	CLPB	TPRG1	CSF2RB	ARID2	
LYRM7	SLAMF6	PSEN1	ST3GAL1	NDRG3	TBL1XR1	TRDMT1	
NAA25	ESRRG	HIST2H2BE	PRKAR2A	DYRK2	RB1	PHF20L1	

Supplementary Table 1. 77 overlapping genes predicted in the intersection of the results of the five miRNA-targets tools

1 Ai, C. & Kong, L. CGPS: A machine learning-based approach integrating multiple gene set analysis tools for better prioritization of biologically relevant pathways. *J Genet Genomics* **45**, 489-504, doi:10.1016/j.jgg.2018.08.002 (2018).

2 Xie, C. *et al.* KOBAS 2.0: a web server for annotation and identification of enriched pathways and diseases. *Nucleic Acids Res* **39**, W316-322, doi:10.1093/nar/gkr483 (2011).
3 Fekete, J. T. & Gyorffy, B. ROCplot.org: Validating predictive biomarkers of chemotherapy/hormonal therapy/anti-HER2 therapy using transcriptomic data of 3,104 breast cancer patients. *Int J Cancer*, doi:10.1002/ijc.32369 (2019).
